# Eating behavior and stress: a pathway to obesity

**DOI:** 10.3389/fpsyg.2014.00434

**Published:** 2014-05-13

**Authors:** Luba Sominsky, Sarah J. Spencer

**Affiliations:** School of Health Sciences and Health Innovations Research Institute, RMIT UniversityMelbourne, VIC, Australia

**Keywords:** corticotropin-releasing hormone (CRH), ghrelin, glucocorticoids, hypothalamic-pituitary-adrenal (HPA) axis, insulin, leptin

## Abstract

Stress causes or contributes to a huge variety of diseases and disorders. Recent evidence suggests obesity and other eating-related disorders may be among these. Immediately after a stressful event is experienced, there is a corticotropin-releasing-hormone (CRH)-mediated suppression of food intake. This diverts the body’s resources away from the less pressing need to find and consume food, prioritizing fight, flight, or withdrawal behaviors so the stressful event can be dealt with. In the hours following this, however, there is a glucocorticoid-mediated stimulation of hunger and eating behavior. In the case of an acute stress that requires a physical response, such as a predator-prey interaction, this hypothalamic-pituitary-adrenal (HPA) axis modulation of food intake allows the stressful event to be dealt with and the energy used to be replaced afterward. In the case of ongoing psychological stress, however, chronically elevated glucocorticoids can lead to chronically stimulated eating behavior and excessive weight gain. In particular, stress can enhance the propensity to eat high calorie “palatable” food via its interaction with central reward pathways. Activation of this circuitry can also interact with the HPA axis to suppress its further activation, meaning not only can stress encourage eating behavior, but eating can suppress the HPA axis and the feeling of stress. In this review we will explore the theme of eating behavior and stress and how these can modulate one another. We will address the interactions between the HPA axis and eating, introducing a potential integrative role for the orexigenic hormone, ghrelin. We will also examine early life and epigenetic modulation of the HPA axis and how this can influence eating behavior. Finally, we will investigate the clinical implications of changes to HPA axis function and how this may be contributing to obesity in our society.

## INTRODUCTION

The body’s stress response is a highly adaptive phenomenon allowing an organism to divert resources to cope with actual or anticipated danger and to restore expended energy that it may fight another day. However, if the stressor is excessive or chronic, responses can become maladaptive. Excessive or chronic stress can trigger or exacerbate a huge variety of diseases and disorders, including mood disorders such as post traumatic stress disorder, anxiety, and depression (McEwen, 2008). Stress, either acute mild stress or prolonged chronic stress, can also influence our appetite, including our drive to eat and the types of food we are likely to select. In this review we will discuss the effects of stress on appetite regulation and how stress may influence our propensity to become obese.

## THE ACUTE EFFECTS OF STRESS ON APPETITE

When an organism encounters a stressful event, a number of steps occur to divert resources appropriately and to assist coping mechanisms (reviewed in, Sapolsky et al., 2000; Papadimitriou and Priftis, 2009). In terms of acute appetite regulation, corticotropin-releasing hormone (CRH) is released from the medial parvocellular (mp) paraventricular nucleus of the hypothalamus (PVN) in response to the stressor. In addition to stimulating adrenocorticotropic hormone (ACTH) release from the pituitary and the cascade of events leading to glucocorticoid release, CRH is also released into the arcuate nucleus of the hypothalamus (ARC) to inhibit neuropeptide Y (NPY)/agouti-related peptide (AGRP) neurons there (Heinrichs et al., 1993; Currie, 2003). This population of cells is normally responsible for stimulating feeding behavior and suppressing energy expenditure; thus CRH released after acute stress inhibits appetite (Heinrichs and Richard, 1999; Richard et al., 2002).

Other molecules from the CRH family, such as urocortins, also play a role in appetite suppression (Weninger et al., 1999; Richard et al., 2002). Thus, early studies from Weninger et al. (1999) showed CRH-deficient mice can have normal stress-induced suppression of food intake, implicating other CRH-like molecules. More recently, Tanaka and colleagues demonstrated both CRH and urocortins suppress food intake, but the urocortins, particularly urocortin 1, do this more effectively (Tanaka et al., 2009). It is likely urocortins 1, 2, and 3 influence appetite suppression by acting on the CRHR2 receptor in the hypothalamus (Richard et al., 2002). Centrally administrated urocortins are also able to suppress ghrelin secretion, potentially preventing ghrelin-induced stimulation of appetite (Yakabi et al., 2011). On the other hand, peripherally administered urocortins act at CRHR2 receptors in the gut to *stimulate* an increase in circulating ghrelin (Wang et al., 2013). These mechanisms likely interact to accurately fine-tune feeding.

In addition to acting on the NPY neurons of the ARC, CRH-induced appetite suppression also involves other regions of the hypothalamus: the PVN, supraoptic nucleus, perifornical and ventromedial hypothalamus; as well as brain regions further afield, the lateral septum, parabrachial nucleus, and the dorsal portion of the anterior bed nucleus of the stria terminalis (BNST; Richard et al., 2002; Ciccocioppo et al., 2003; Fatima et al., 2013). Thus, CRH injected directly into the dorsal anterior BNST (but not the ventral part or other brain regions such as the central amygdala or locus coeruleus) significantly reduces food intake in already food-deprived rats (Ciccocioppo et al., 2003).

## THE CHRONIC EFFECTS OF STRESS ON APPETITE

Ethologically, the appetite suppressive response is useful for diverting energy away from food-seeking behavior and eating toward more pressing concerns, such as escaping the predator or rehearsing the speech. With longer-term stressors, however, the energy used coping needs to be replaced. In the hours to days after the onset of an ongoing stressful event (e.g., infection, bereavement), glucocorticoids in the bloodstream are elevated. Peripherally, glucocorticoids enhance the activity of lipoprotein lipase in adipose tissue, leading to an increase in fat storage (Bjorntorp, 1996, 2001). This occurs particularly in visceral fat where lipoprotein lipase activity is higher (Marin et al., 1992a). Thus, chronically elevated glucocorticoids contribute to visceral fat accumulation (Marin et al., 1992b; Rosmond et al., 1998; Epel et al., 2000). Other mechanisms by which glucocorticoids stimulate excess fat deposition are reviewed in (Spencer and Tilbrook, 2011).

In terms of feeding behavior, glucocorticoids also act on the hypothalamus to stimulate appetite (Santana et al., 1995; Dallman et al., 2004). Thus, in humans, a peripheral injection of CRH leads to increased food intake 1 h later but the amount of food consumed is directly correlated with the magnitude of the cortisol response to the injection (George et al., 2010). Glucocorticoids stimulate food intake by interacting with several appetite-regulating targets. They increase AMP-activated protein kinase signaling in the ARC to up-regulate NPY and AGRP expression in this region and stimulate the actions of these orexigenic peptides (Savontaus et al., 2002; Konno et al., 2008; Shimizu et al., 2008). Glucocorticoids also influence the function of leptin, whose normal role is to signal satiety thus suppressing appetite. Although glucocorticoids stimulate leptin release from adipose tissue, which would normally lead to appetite suppression, they also reduce the sensitivity of the brain to leptin, contributing to leptin resistance (Zakrzewska et al., 1997, 1999; Jequier, 2002). Thus, adrenalectomized rats respond to intracerebroventricular (icv) leptin with a larger reduction in food intake and body weight than intact rats and the addition of glucocorticoids reduces leptin’s anorexigenic effects (Zakrzewska et al., 1997).

Insulin is another appetite-regulatory hormone that is influenced by glucocorticoids, although the role of glucocorticoids here is more complex. Insulin usually acts at the hypothalamus to reduce food intake and at the ventral tegmental area (VTA) to reduce the dopaminergic neuron-mediated rewarding nature of food (Figlewicz et al., 2008). Acutely, glucocorticoids stimulate insulin secretion from the pancreas (Strack et al., 1995), having an appetite-suppressant effect. However, chronically activated glucocorticoids also contribute to insulin resistance. Thus, as is seen with leptin, glucocorticoids contribute to a reduced ability of insulin to inhibit NPY/AGRP neurons in the ARC, which has the converse effect of lessening appetite suppression (Asensio et al., 2004). The intermediate role of glucocorticoids in the connection between insulin sensitivity and increased appetite is typically observed in patients with Cushing’s syndrome. Glucocorticoid excess in these patients leads to an increase in appetite, weight gain and insulin resistance (Anagnostis et al., 2009).

Glucocorticoids also influence food intake by enhancing the preference for “comfort foods.” Insulin’s suppressive effect on reward pathways likely means the food needs to be more “rewarding” to achieve the same effect; hence under stressed conditions rats prefer foods that are high in fat and sucrose when a choice is available (la Fleur et al., 2004; Warne et al., 2006, 2009). Chronically stressed animals thus prefer calorically dense foods (Pecoraro et al., 2004; Foster et al., 2009). This enhanced caloric intake has been proposed to correspond with the increased brain energy demand and thus preferential glucose allocation to the brain under the conditions of stress (Peters et al., 2011). Remarkably, this highly palatable food also leads to a reward-mediated negative feedback onto the hypothalamic-pituitary-adrenal (HPA) axis to suppress it. In this way, a junk food diet or a stress-induced ice-cream binge may actually alleviate the symptoms of stress (Pecoraro et al., 2004; Foster et al., 2009). Rats given chronic restraint stress for 3 h per day for 5 days voluntarily eat more lard and sucrose than control rats, and the plasma ACTH and glucocorticoid response to this restraint is suppressed in those rats that were given free access to these “comfort” foods. Unsurprisingly, these rats also become heavier than their restraint-stressed counterparts given normal chow (Pecoraro et al., 2004).

Another mechanism by which glucocorticoids can influence appetite during stress is via its interaction with ghrelin. Ghrelin is a peptide derived principally from the gut. It is released as a signal of hunger or just prior to the usual meal time to stimulate feeding (Hosoda et al., 2006). Circulating ghrelin is increased in response to stress (Kristenssson et al., 2006) and probably acts at the level of the anterior pituitary as well as higher brain regions, such as the centrally projecting Edinger Westphal nucleus (EWcp), to modulate ACTH release from the pituitary and regulate glucocorticoid negative feedback (Spencer et al., 2012). Chronic or severe stress resulting in elevated glucocorticoid secretion will also lead to elevated circulating ghrelin levels, culminating in increased ghrelin-mediated stimulation of NPY/AGRP and increased food intake (Asakawa et al., 2001; Kristenssson et al., 2006; Lutter et al., 2008; Ochi et al., 2008). Interestingly, while stress-induced elevation of ghrelin corresponds with exacerbation of social avoidance and increased food intake in wild-type animals, deletion of ghrelin receptor (growth hormone secretagogue receptor; *GHSR*-/-) results in even more pronounced social avoidance than stress does, but it does not increase food intake (Lutter et al., 2008). Activation of ghrelin signaling in response to stress may thus represent a coping mechanism, where combatting the effects of the stressor is prioritized at the expense of increased food intake. Acute psychosocial stress in human subjects has been also documented to induce increased release of ghrelin (Rouach et al., 2007).

The consequences of a chronically stimulated HPA axis response to stress are easy to imagine. Excessive glucocorticoid production and/or elevated basal glucocorticoids, as can occur with chronic stress and mood disorders (McEwen, 2008; Lupien et al., 2009), leads to energy conservation and appetite stimulation. Excessive high calorie foods are consumed and excess weight gain and eventually obesity ensue (De Vriendt et al., 2009). However, exposure to chronic stress may also suppress appetite in some individuals, particularly in unrestrained eaters, as opposed to restrained eaters who voluntarily restrict their diet to maintain proper weight, but tend to increase their food intake when stressed (Greeno and Wing, 1994). Depression, which can often be triggered by chronic exposure to stressful events, is also frequently associated with reduced appetite (Nestler et al., 2002). It is likely ghrelin plays a principal role in determining if an individual responds to stress with an increase or a decrease in appetite. Individuals classified as “emotional eaters” (those who consume more highly palatable food during stress) have lower basal ghrelin than “non-emotional eaters” (those whose food intake is suppressed or unchanged by stress; Raspopow et al., 2010). Lower basal ghrelin levels are also associated with binge-eating, an emotional eating disorder (Geliebter et al., 2005). Stress-induced ghrelin levels remain unaltered by food intake in emotional eaters but are rapidly restored to baseline by food in non-emotional eaters (Raspopow et al., 2014). Thus, emotional eaters may require relatively more palatable food to suppress stress-induced ghrelin to the same degree as non-emotional eaters.

## EARLY LIFE HPA AXIS DEVELOPMENT AND ITS EFFECTS ON EATING BEHAVIOR

Lifetime experience, whether acute or chronic, clearly shapes both HPA axis and eating behavior. However, how an individual responds to each experience can be influenced at times outside the immediately pertinent event. It is now well accepted that the early life period is one of significant vulnerability to programming influences. For instance, central pathways governing feeding and metabolism start to develop at specific stages of early life and, at this time, the animal is particularly vulnerable to influences from the environment.

An initial critical window of vulnerability occurs in prenatal life, when HPA axis and feeding-regulatory pathways begin to develop. For instance, both stress (or synthetic glucocorticoids) and poor nutrition *in utero* can have significant long-term consequences for feeding and behavior. Excessive stress during pregnancy can lead to HPA axis dysfunction (Henry et al., 1994; Rossi-George et al., 2009) and a long-term susceptibility to mood disorders in the offspring (Vallee et al., 1997), as well as impaired learning and memory (Lordi et al., 1997; Entringer et al., 2009), changes to reward pathways that lead to addictive behaviors (Morley-Fletcher et al., 2004; Thomas et al., 2009), and also, obesity (Li et al., 2010). The effects of prenatal stress on long-term feeding biology have been elegantly reviewed in (Entringer et al., 2012; Entringer and Wadhwa, 2013). Conversely, obesity during pregnancy, or even a pregnancy diet high in fat and sugar, can influence metabolic phenotype long-term as well as central reward processing, altering the way the rewarding aspects of food are perceived throughout life, leading to a preference for fatty, sugary foods (Ong and Muhlhausler, 2011).

This type of vulnerability in the developing individual continues postnatally.

In the rodent the hypothalamic connectivity involved in feeding develops during the second week postnatally (Bouret et al., 2004a,b). Leptin is one critical trophic factor in stimulating this growth. Thus, insufficient leptin available in the dam’s milk while these pathways are developing can disrupt the formation of these connections (Bouret and Simerly, 2007). A premature leptin surge or excessive leptin, such as can occur with *in utero* growth restriction or with obese or hyperleptinemic dams, can also disrupt this connectivity and result in a subsequent insensitivity to satiety signals (Yura et al., 2005; Kirk et al., 2009). Similarly, ghrelin normally counteracts leptin’s trophic effects on these regions and a change in the timing or magnitude of the expected progressive elevation in plasma ghrelin can also disrupt this development (Grove and Cowley, 2005). The ultimate effect of such developmental influences on the animal is a disruption of central responses to nutritional status and disrupted feeding behavior.

It is interesting to note that development of the HPA axis occurs in the rodent at similar times to the development of feeding-regulatory pathways. An animal’s ability to respond to stress is immature at birth and the lifespan is characterized by a stress-hyporesponsive period that lasts from approximately the first to second weeks of life (Sapolsky and Meaney, 1986). Excessive stress, exposure to glucocorticoids, or prolonged absence from the dam can permanently terminate this stress hyporesponsive period, leading to life-long hypersensitivity to stress (Lehmann et al., 2002a,b; Barna et al., 2003; Xu et al., 2011). Certainly, early life stressful events such as maternal separation in the rodent, or child abuse/loss of a parent in humans can cause disruption of the HPA axis in this way (Koch et al., 2008; D’Argenio et al., 2009). However, neonatal developmental influences can also be fairly subtle and still have pronounced effects. For instance, Meaney’s group has shown rat pups given high-intensity nursing and grooming by their dams grow up to have attenuated HPA axis responses to psychological stress and reduced vulnerability to anxiety (Liu et al., 1997; Champagne and Meaney, 2001).

In addition to, or perhaps as a result of, disrupting the HPA axis, the parental influence at this time is also crucial for establishing feeding patterns long-term. Thus, maternal separation can lead to the offspring having lower voluntary food intake and a preference for foods low in carbohydrates (Penke et al., 2001), while social isolation in previously maternally separated rats elevates food intake and weight gain (Ryu et al., 2009). It is likely this effect of the early environment on feeding patterns long-term is somewhat adaptive for the animal. Thus, early maternal separation in the wild rat likely occurs when food is scarce and foraging difficult. Thus, the offspring is brought into a world of food scarcity and high stress and its physiology adjusts accordingly to become hypersensitive to the effects of stress and to overeat. Essentially the neonatal environment thus imposes a drive to make the most of feeding opportunities when they are available (Meaney, 2001).

## MECHANISMS OF EARLY LIFE INFLUENCE ON HPA AXIS FUNCTION

**FIGURE 1:**
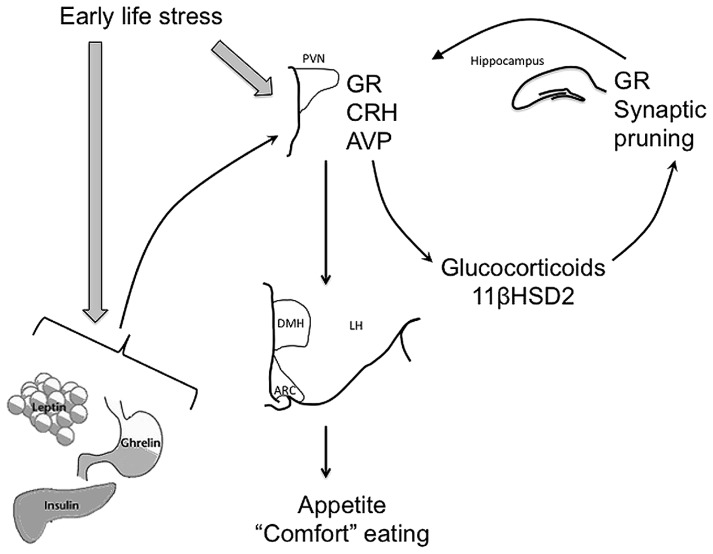
**Early life stress can influence development of the HPA axis, as well as regulation of satiety-related hormones, leptin, insulin, and ghrelin to alter feeding behavior long-term.** Thus, early life stress can lead to epigenetic modification of glucocorticoid receptor (GR) expression in the hypothalamus and hippocampus and arginine vasopressin (AVP) and corticotropin-releasing hormone (CRH) in the hypothalamus, resulting in suppressed GR and increased AVP and CRH activity in response to stress later in life. Synaptic pruning in the hippocampus and circulating 11βHSD2 are also affected leading to elevated circulating glucocorticoid (GC) concentrations both under basal conditions and in response to stress. These effects of early life stress are ultimately seen in altered outputs from the paraventricular nucleus of the hypothalamus (PVN) to feeding-related nuclei such as the arcuate nucleus (ARC) and the dorsomedial nucleus of the hypothalamus (DMH). Early life stress can also potentially induce increased release of trophic/satiety hormones such as leptin, insulin, and ghrelin, again influencing appetite, feeding behavior, and metabolism throughout life.

Early life events are able to disrupt HPA axis function in a variety of ways (**Figure 1**). Prior to birth, the fetus is remarkably well protected from the effects of stress. The placenta produces 11β hydroxysteroid dehydrogenase 2 (11βHSD2), which converts active glucocorticoids from the mother into the inactive form, ensuring maternal glucocorticoids are prevented from reaching fetal circulation (Lucassen et al., 2009). Central changes also occur in the mother to ensure she responds to stress by secreting less glucocorticoids; for instance, allopregnanolone-mediated inhibition of the noradrenergic input to the PVN is enhanced as progesterone levels increase with pregnancy, meaning HPA axis activation is suppressed (Brunton et al., 2005, 2009). However, severe or prolonged stress or synthetic glucocorticoid exposure can over-ride these protective mechanisms and influence the development of the fetal HPA axis. For instance, excess maternal glucocorticoids can increase fetal circulating glucocorticoid levels and can alter fetal 11βHSD2 (Clifton et al., 2006) and glucocorticoid receptor (GR) expression (Edwards et al., 1993). Excess fetal glucocorticoids can also interfere with normal brain growth and development at this time, with restraint stress to the dam during pregnancy leading to reduced levels of proteins such as growth-associated protein of 43 kDa (GAP-43) that are involved in synaptic pruning (Pfenninger et al., 1991; Larsson, 2006; Jutapakdeegul et al., 2009).

Postnatally there are fewer mechanisms to protect the animal from the effects of stress and excessive glucocorticoids. The presence of the dam, in rodents, is essential for the maintenance of attenuated sensitivity to stress in the stress hypo-responsive period, but the neonatal HPA axis is still very vulnerable at this time. As with fetal glucocorticoids, postnatal glucocorticoids or stress can alter synaptic pruning and can also lead to reduced GR expression in brain regions important for glucocorticoid negative feedback, the hypothalamus and hippocampus (Liu et al., 1997).

These effects of the perinatal environment on GR can be imposed long-term via changes to the epigenome. For instance, even something as subtle as the style of attention imparted by the dam to her offspring can induce pronounced epigenetic changes to GR expression. When rat pups are groomed by the dam it induces a rise, in the pup, of nerve growth factor inducible factor A (NGFI-A) expression (Hellstrom et al., 2012). The increase in NGFI-A expression in turn leads to increases in histone acetylation of the GR, demethylation of the GR promoter and increased GR activity (Hellstrom et al., 2012). Thus, pups that experienced a paucity of grooming in early life have reduced NGFI-A expression and suppressed GR activity and expression in glucocorticoid negative feedback regions. The long-term effect of this early under-grooming is a hypersensitivity to the effects of stress (Champagne and Meaney, 2001). Elevations in GR expression due to early life influence have also been linked to excess weight gain throughout life (Stevens et al., 2010; Begum et al., 2012).

Arginine vasopressin (AVP) regulation of the HPA axis response to stress is also subject to epigenetic modification by early life events. Thus, in the mouse, early separation from the dam leads to changes in DNA methylation, resulting in increased PVN AVP expression and changes in coping responses to stress (Murgatroyd et al., 2009; Murgatroyd and Spengler, 2011). While the early life period is one of particular vulnerability to environmental influences, epigenetic modification can occur in response to the environment at any time. Thus, chronic social stress in adult mice can induce lasting demethylation of the CRH gene, resulting in heightened anxiety-like behavior (Elliott et al., 2010).

In addition to the early influence of stress and glucocorticoids directly on the HPA axis, stress and glucocorticoids can also independently influence development of the feeding circuitry discussed above. For instance, perinatal glucocorticoids, in rodents and humans, can lead to elevations in plasma leptin (Bruder et al., 2005; Marinoni et al., 2008). Given what we know about the sensitivity of the developing hypothalamic connectivity to circulating leptin at this time, it is highly likely this glucocorticoid-mediated increase in leptin interferes with the normal leptin-induced establishment of connections between the ARC, PVN, dorsomedial nucleus of the hypothalamus (DMH), and lateral hypothalamus (LH). Glucocorticoids can also influence levels of other crucial trophic hormones at this time, increasing insulin release from the pancreas (Moyer-Mileur et al., 2011) and ghrelin release from the gut (Hosoda et al., 2006; Kristenssson et al., 2006). There is even recent evidence maternal insulin sensitivity during pregnancy can influence fetal brain activity and may contribute to prenatal programming of long-term insulin sensitivity (Linder et al., 2014). Again, it is likely these changes are able to interfere with appropriate establishment of feeding-related circuitry in the hypothalamus. It is also worth noting these trophic factors may also contribute to HPA axis development, further consolidating the link between the HPA axis and feeding. Thus, elevated neonatal leptin levels (independent of other environmental stimuli) can lead to an increase in GR in the hypothalamus and hippocampus and resulting changes in HPA axis sensitivity to glucocorticoid negative feedback (Proulx et al., 2001).

## CONCLUSION AND CLINICAL IMPLICATIONS

The discussed data make it clear that the HPA, stress, axis and feeding regulation are inextricably linked, with the early life developmental environment being critical in establishing both. The challenge now will be to ensure we achieve the appropriate balance when influencing these systems with parental care and neonatal medical treatments. There is no doubt that several current perinatal treatments, while crucial for their immediate purpose, have far-reaching side-effects on systems such as the HPA axis and feeding circuitry. For instance, synthetic glucocorticoid, administered prenatally to assist in lung development, may elevate plasma leptin (Marinoni et al., 2008), stimulate epigenetic modifications in GR and elevate 11βHSD2 (Clifton et al., 2006). Similarly, the current practice of intensively feeding premature and small for gestational age babies to accelerate brain and lung development has the negative side-effect of predisposing these babies to long-term excess weight gain (Ong et al., 2000; Stettler et al., 2005). While these strategies may be essential in the immediate term to ensure the newborn’s survival, consideration should be given to how we can mitigate the long-term negative effects. Understanding of the mechanisms by which stress interacts with eating behavior in the developed adult is also essential for behavioral and pharmaceutical treatments to prevent excess weight gain in at-risk patients.

## AUTHOR CONTRIBUTIONS

Luba Sominsky and Sarah J. Spencer conceived of, researched, drafted, and finalized this review. They give final approval of the version to be published and agree to be accountable for all aspects of the work in ensuring that questions related to the accuracy or integrity of any part of the work are appropriately investigated and resolved.

## Conflict of Interest Statement

The authors declare that the research was conducted in the absence of any commercial or financial relationships that could be construed as a potential conflict of interest.

## References

[B1] Anagnostis P. Athyros V. G. Tziomalos K. Karagiannis A. Mikhailidis D. P. (2009). Clinical review: the pathogenetic role of cortisol in the metabolic syndrome: a hypothesis. *J. Clin. Endocrinol. Metab.* 94 2692–2701 10.1210/jc.2009-0370 19470627

[B2] Asakawa A. Inui A. Kaga T. Yuzuriha H. Nagata T. Fujimiya M. (2001). A role of ghrelin in neuroendocrine and behavioral responses to stress in mice. *Neuroendocrinology* 74 143–147 10.1159/000054680 11528215

[B3] Asensio C. Muzzin P. Rohner-Jeanrenaud F. (2004). Role of glucocorticoids in the physiopathology of excessive fat deposition and insulin resistance. *Int. J. Obes. Relat. Metab. Disord.* 28(Suppl. 4) S45–S52 10.1038/sj.ijo.0802856 15592486

[B4] Barna I. Balint E. Baranyi J. Bakos N. Makara G. B. Haller J. (2003). Gender-specific effect of maternal deprivation on anxiety and corticotropin-releasing hormone mRNA expression in rats. *Brain Res. Bull.* 62 85–91 10.1016/S0361-9230(03)00216-8 14638381

[B5] Begum G. Stevens A. Smith E. B. Connor K. Challis J. R. Bloomfield F. (2012). Epigenetic changes in fetal hypothalamic energy regulating pathways are associated with maternal undernutrition and twinning. *FASEB J.* 26 1694–1703 10.1096/fj.11-198762 22223754 PMC3316895

[B6] Bjorntorp P. (1996). The regulation of adipose tissue distribution in humans. *Int. J. Obes. Relat. Metab. Disord.* 20 291–302 8680455

[B7] Bjorntorp P. (2001). Do stress reactions cause abdominal obesity and comorbidities? *Obes. Rev.* 2 73–86 10.1046/j.1467-789x.2001.00027.x 12119665

[B8] Bouret S. G. Draper S. J. Simerly R. B. (2004a). Formation of projection pathways from the arcuate nucleus of the hypothalamus to hypothalamic regions implicated in the neural control of feeding behavior in mice. *J. Neurosci.* 24 2797–2805 10.1523/JNEUROSCI.5369-03.2004 15028773 PMC6729527

[B9] Bouret S. G. Draper S. J. Simerly R. B. (2004b). Trophic action of leptin on hypothalamic neurons that regulate feeding. *Science* 304 108–110 10.1126/science.1095004 15064420

[B10] Bouret S. G. Simerly R. B. (2007). Development of leptin-sensitive circuits. *J. Neuroendocrinol.* 19 575–582 10.1111/j.1365-2826.2007.01563.x 17620099

[B11] Bruder E. D. Jacobson L. Raff H. (2005). Plasma leptin and ghrelin in the neonatal rat: interaction of dexamethasone and hypoxia. *J. Endocrinol.* 185 477–484 10.1677/joe.1.06159 15930174 PMC1249478

[B12] Brunton P. J. McKay A. J. Ochedalski T. Piastowska A. Rebas E. Lachowicz A. (2009). Central opioid inhibition of neuroendocrine stress responses in pregnancy in the rat is induced by the neurosteroid allopregnanolone. *J. Neurosci.* 29 6449–6460 10.1523/JNEUROSCI.0708-09.2009 19458216 PMC6665894

[B13] Brunton P. J. Meddle S. L. Ma S. Ochedalski T. Douglas A. J. Russell J. A. (2005). Endogenous opioids and attenuated hypothalamic-pituitary-adrenal axis responses to immune challenge in pregnant rats. *J. Neurosci.* 25 5117–5126 10.1523/JNEUROSCI.0866-05.2005 15917452 PMC6724825

[B14] Champagne F. Meaney M. J. (2001). Like mother, like daughter: evidence for non-genomic transmission of parental behavior and stress responsivity. *Prog. Brain Res.* 133 287–302 10.1016/S0079-6123(01)33022-4 11589138

[B15] Ciccocioppo R. Fedeli A. Economidou D. Policani F. Weiss F. Massi M. (2003). The bed nucleus is a neuroanatomical substrate for the anorectic effect of corticotropin-releasing factor and for its reversal by nociceptin/orphanin FQ. *J. Neurosci.* 23 9445–9451 14561874 10.1523/JNEUROSCI.23-28-09445.2003 PMC3035815

[B16] Clifton V. L. Rennie N. Murphy V. E. (2006). Effect of inhaled glucocorticoid treatment on placental 11β-hydroxysteroid dehydrogenase type 2 activity and neonatal birthweight in pregnancies complicated by asthma. *Aust. N. Z. J. Obstet. Gynaecol.* 46 136–140 10.1111/j.1479-828X.2006.00543.x 16638036

[B17] Currie P. J. (2003). Integration of hypothalamic feeding and metabolic signals: focus on neuropeptide Y. *Appetite* 41 335–337 10.1016/j.appet.2003.08.011 14637334

[B18] Dallman M. F. la Fleur S. E. Pecoraro N. C. Gomez F. Houshyar H. Akana S. F. (2004). Minireview: glucocorticoids–food intake, abdominal obesity, and wealthy nations in 2004. *Endocrinology* 145 2633–2638 10.1210/en.2004-0037 15044359

[B19] D’Argenio A. Mazzi C. Pecchioli L. Di Lorenzo G. Siracusano A. Troisi A. (2009). Early trauma and adult obesity: is psychological dysfunction the mediating mechanism? *Physiol. Behav.* 98 543–546 10.1016/j.physbeh.2009.08.010 19733190

[B20] De Vriendt T. Moreno L. A De Henauw S. (2009). Chronic stress and obesity in adolescents: scientific evidence and methodological issues for epidemiological research. *Nutr. Metab. Cardiovasc. Dis.* 19 511–519 10.1016/j.numecd.2009.02.009 19362453

[B21] Edwards C. R. Benediktsson R. Lindsay R. S. Seckl J. R. (1993). Dysfunction of placental glucocorticoid barrier: link between fetal environment and adult hypertension? *Lancet* 341 355–357 8094124 10.1016/0140-6736(93)90148-a

[B22] Elliott E. Ezra-Nevo G. Regev L. Neufeld-Cohen A. Chen A. (2010). Resilience to social stress coincides with functional DNA methylation of the Crf gene in adult mice. *Nat. Neurosci.* 13 1351–1353 10.1038/nn.2642 20890295

[B23] Entringer S. Buss C. Kumsta R. Hellhammer D. H. Wadhwa P. D. Wust S. (2009). Prenatal psychosocial stress exposure is associated with subsequent working memory performance in young women. *Behav. Neurosci.* 123 886–893 10.1037/a0016265 19634949 PMC2862630

[B24] Entringer S. Buss C. Swanson J. M. Cooper D. M. Wing D. A. Waffarn F. (2012). Fetal programming of body composition, obesity, and metabolic function: the role of intrauterine stress and stress biology. *J. Nutr. Metab.* 2012 632548 10.1155/2012/632548 PMC3359710 22655178

[B25] Entringer S. Wadhwa P. D. (2013). Developmental programming of obesity and metabolic dysfunction: role of prenatal stress and stress biology. *Nestle Nutr. Inst. Workshop Ser.* 74 107–120 10.1159/000348454 23887109 PMC4159714

[B26] Epel E. S. McEwen B. Seeman T. Matthews K. Castellazzo G. Brownell K. D. (2000). Stress and body shape: stress-induced cortisol secretion is consistently greater among women with central fat. *Psychosom. Med.* 62 623–632 11020091 10.1097/00006842-200009000-00005

[B27] Fatima A. Andrabi S. Wolf G. Engelmann M. Spina M. G. (2013). Urocortin 1 administered into the hypothalamic supraoptic nucleus inhibits food intake in freely fed and food-deprived rats. *Amino Acids* 44 879–885 10.1007/s00726-012-1415-7 23076252

[B28] Figlewicz D. P. Bennett J. L. Aliakbari S. Zavosh A. Sipols A. J. (2008). Insulin acts at different CNS sites to decrease acute sucrose intake and sucrose self-administration in rats. *Am. J. Physiol. Regul. Integr. Comp. Physiol.* 295 R388–R394 10.1152/ajpregu.90334.2008 18525010 PMC2519924

[B29] Foster M. T. Warne J. P. Ginsberg A. B. Horneman H. F. Pecoraro N. C. Akana S. F. (2009). Palatable foods, stress, and energy stores sculpt corticotropin-releasing factor, adrenocorticotropin, and corticosterone concentrations after restraint. *Endocrinology* 150 2325–2333 10.1210/en.2008-1426 19106219 PMC2671911

[B30] Geliebter A. Gluck M. E. Hashim S. A. (2005). Plasma ghrelin concentrations are lower in binge-eating disorder. *J. Nutr.* 135 1326–1330 15867334 10.1093/jn/135.5.1326

[B31] George S. A. Khan S. Briggs H. Abelson J. L. (2010). CRH-stimulated cortisol release and food intake in healthy, non-obese adults. *Psychoneuroendocrinology* 35 607–612 10.1016/j.psyneuen.2009.09.017 19828258 PMC2843773

[B32] Greeno C. G. Wing R. R. (1994). Stress-induced eating. *Psychol. Bull.* 115 444–464 10.1037/0033-2909.115.3.444 8016287

[B33] Grove K. L. Cowley M. A. (2005). Is ghrelin a signal for the development of metabolic systems? *J. Clin. Invest.* 115 3393–3397 10.1172/JCI27211 16322785 PMC1297272

[B34] Heinrichs S. C. Menzaghi F. Pich E. M. Hauger R. L. Koob G. F. (1993). Corticotropin-releasing factor in the paraventricular nucleus modulates feeding induced by neuropeptide Y. *Brain Res.* 611 18–24 10.1016/0006-8993(93)91771-J 8518948

[B35] Heinrichs S. C. Richard D. (1999). The role of corticotropin-releasing factor and urocortin in the modulation of ingestive behavior. *Neuropeptides* 33 350–359 10.1054/npep.1999.0047 10657512

[B36] Hellstrom I. C. Dhir S. K. Diorio J. C. Meaney M. J. (2012). Maternal licking regulates hippocampal glucocorticoid receptor transcription through a thyroid hormone-serotonin-NGFI-A signalling cascade. *Philos. Trans. R. Soc. Lond. B Biol. Sci.* 367 2495–2510 10.1098/rstb.2012.0223 22826348 PMC3405683

[B37] Henry C. Kabbaj M. Simon H. Le Moal M. Maccari S. (1994). Prenatal stress increases the hypothalamo-pituitary-adrenal axis response in young and adult rats. *J. Neuroendocrinol.* 6 341–345 10.1111/j.1365-2826.1994.tb00591.x 7920600

[B38] Hosoda H. Kojima M. Kangawa K. (2006). Biological, physiological, and pharmacological aspects of ghrelin. *J. Pharmacol. Sci.* 100 398–410 10.1254/jphs.CRJ06002X 16612045

[B39] Jequier E. (2002). Leptin signaling, adiposity, and energy balance. *Ann. N. Y. Acad. Sci.* 967 379–388 10.1111/j.1749-6632.2002.tb04293.x 12079865

[B40] Jutapakdeegul N. Polboon N. Afadlal S. Phansuwan-Pujito P. Govitrapong P. (2009). Repeated restraint stress and corticosterone injections during late pregnancy alter GAP-43 expression in the hippocampus and prefrontal cortex of rat pups. *Int. J. Dev. Neurosci.* 28 83–90 10.1016/j.ijdevneu.2009.09.003 19782125

[B41] Kirk S. L. Samuelsson A. M. Argenton M. Dhonye H. Kalamatianos T. Poston L. (2009). Maternal obesity induced by diet in rats permanently influences central processes regulating food intake in offspring. *PLoS ONE* 4:e5870. 10.1371/journal.pone.0005870 PMC2690656 19516909

[B42] Koch F. S. Sepa A. Ludvigsson J. (2008). Psychological stress and obesity. *J. Pediatr.* 153 839–844 10.1016/j.jpeds.2008.06.016 18657829

[B43] Konno J. Yoshida S. Ina A. Ohmomo H. Shutoh F. Nogami H. (2008). Upregulated expression of neuropeptide Y in hypothalamic-pituitary system of rats by chronic dexamethasone administration. *Neurosci. Res.* 60 259–265 10.1016/j.neures.2007.11.005 18164503

[B44] Kristenssson E. Sundqvist M. Astin M. Kjerling M. Mattsson H. Dornonville de la Cour C. (2006). Acute psychological stress raises plasma ghrelin in the rat. *Regul. Pept.* 134 114–117 10.1016/j.regpep.2006.02.003 16540188

[B45] la Fleur S. E. Akana S. F. Manalo S. L. Dallman M. F. (2004). Interaction between corticosterone and insulin in obesity: regulation of lard intake and fat stores. *Endocrinology* 145 2174–2185 10.1210/en.2003-1359 14962993

[B46] Larsson C. (2006). Protein kinase C and the regulation of the actin cytoskeleton. *Cell. Signal.* 18 276–284 10.1016/j.cellsig.2005.07.010 16109477

[B47] Lehmann J. Pryce C. R. Jongen-Relo A. L. Stohr T. Pothuizen H. H. Feldon J. (2002a). Comparison of maternal separation and early handling in terms of their neurobehavioral effects in aged rats. *Neurobiol. Aging* 23 457–466 10.1016/S0197-4580(01)00320-7 11959408

[B48] Lehmann J. Russig H. Feldon J. Pryce C. R. (2002b). Effect of a single maternal separation at different pup ages on the corticosterone stress response in adult and aged rats. *Pharmacol. Biochem. Behav.* 73 141–145 10.1016/S0091-3057(02)00788-8 12076733

[B49] Li J. Olsen J. Vestergaard M. Obel C. Baker J. L. Sorensen T. I. (2010). Prenatal stress exposure related to maternal bereavement and risk of childhood overweight. *PLoS ONE* 5:e11896. 10.1371/journal.pone.0011896 PMC2912844 20689593

[B50] Linder K. Schleger F. Ketterer C. Fritsche L. Kiefer-Schmidt I. Hennige A. (2014). Maternal insulin sensitivity is associated with oral glucose-induced changes in fetal brain activity. *Diabetologia* 10.1007/s00125-014-3217-9 [Epub ahead of print] 24671273

[B51] Liu D. Diorio J. Tannenbaum B. Caldji C. Francis D. Freedman A. (1997). Maternal care, hippocampal glucocorticoid receptors, and hypothalamic-pituitary-adrenal responses to stress. *Science* 277 1659–1662 10.1126/science.277.5332.1659 9287218

[B52] Lordi B. Protais P. Mellier D. Caston J. (1997). Acute stress in pregnant rats: effects on growth rate, learning, and memory capabilities of the offspring. *Physiol. Behav.* 62 1087–1092 10.1016/S0031-9384(97)00261-8 9333204

[B53] Lucassen P. J. Bosch O. J. Jousma E. Kromer S. A. Andrew R. Seckl J. R. (2009). Prenatal stress reduces postnatal neurogenesis in rats selectively bred for high, but not low, anxiety: possible key role of placental 11β-hydroxysteroid dehydrogenase type 2. *Eur. J. Neurosci.* 29 97–103 10.1111/j.1460-9568.2008.06543.x 19032587

[B54] Lupien S. J. McEwen B. S. Gunnar M. R. Heim C. (2009). Effects of stress throughout the lifespan on the brain, behaviour and cognition. *Nat. Rev. Neurosci.* 10 434–445 10.1038/nrn2639 19401723

[B55] Lutter M. Sakata I. Osborne-Lawrence S. Rovinsky S. A. Anderson J. G. Jung S. (2008). The orexigenic hormone ghrelin defends against depressive symptoms of chronic stress. *Nat. Neurosci.* 11 752–753 10.1038/nn.2139 18552842 PMC2765052

[B56] Marin P. Andersson B. Ottosson M. Olbe L. Chowdhury B. Kvist H. (1992a). The morphology and metabolism of intraabdominal adipose tissue in men. *Metabolism* 41 1242–1248 10.1016/0026-0495(92)90016-4 1435298

[B57] Marin P. Darin N. Amemiya T. Andersson B. Jern S. Bjorntorp P. (1992b). Cortisol secretion in relation to body fat distribution in obese premenopausal women. *Metabolism* 41 882–886 10.1016/0026-0495(92)90171-6 1640867

[B58] Marinoni E. Letizia C. Ciardo F. Corona G. Moscarini M Di Iorio R. (2008). Effects of prenatal betamethasone administration on leptin and adiponectin concentrations in maternal and fetal circulation. *Am. J. Obstet. Gynecol.* 199 141.e1–141.e6. 10.1016/j.ajog.2008.02.047 18456235

[B59] McEwen B. S. (2008). Central effects of stress hormones in health and disease: understanding the protective and damaging effects of stress and stress mediators. *Eur. J. Pharmacol.* 583 174–185 10.1016/j.ejphar.2007.11.071 18282566 PMC2474765

[B60] Meaney M. J. (2001). Maternal care, gene expression, and the transmission of individual differences in stress reactivity across generations. *Annu. Rev. Neurosci.* 24 1161–1192 10.1146/annurev.neuro.24.1.1161 11520931

[B61] Morley-Fletcher S. Puopolo M. Gentili S. Gerra G. Macchia T. Laviola G. (2004). Prenatal stress affects 3,4-methylenedioxymethamphetamine pharmacokinetics and drug-induced motor alterations in adolescent female rats. *Eur. J. Pharmacol.* 489 89–92 10.1016/j.ejphar.2004.02.028 15063159

[B62] Moyer-Mileur L. J. Haley S. Gulliver K. Thomson A. Slater H. Barrett B. (2011). Mechanical-tactile stimulation (MTS) during neonatal stress prevents hyperinsulinemia despite stress-induced adiposity in weanling rat pups. *Early Hum. Dev.* 87 159–163 10.1016/j.earlhumdev.2010.12.001 21211914 PMC3228309

[B63] Murgatroyd C. Patchev A. V. Wu Y. Micale V. Bockmuhl Y. Fischer D. (2009). Dynamic DNA methylation programs persistent adverse effects of early-life stress. *Nat. Neurosci.* 12 1559–1566 10.1038/nn.2436 19898468

[B64] Murgatroyd C. Spengler D. (2011). Epigenetic programming of the HPA axis: early life decides. *Stress* 14 581–589 10.3109/10253890.2011.602146 21854166

[B65] Nestler E. J. Barrot M. DiLeone R. J. Eisch A. J. Gold S. J. Monteggia L. M. (2002). Neurobiology of depression. *Neuron* 34 13–25 10.1016/S0896-6273(02)00653-0 11931738

[B66] Ochi M. Tominaga K. Tanaka F. Tanigawa T. Shiba M. Watanabe T. (2008). Effect of chronic stress on gastric emptying and plasma ghrelin levels in rats. *Life Sci.* 82 862–868 10.1016/j.lfs.2008.01.020 18343456

[B67] Ong K. K. Ahmed M. L. Emmett P. M. Preece M. A. Dunger D. B. (2000). Association between postnatal catch-up growth and obesity in childhood: prospective cohort study. *BMJ* 320 967–971 10.1136/bmj.320.7240.967 10753147 PMC27335

[B68] Ong Z. Y. Muhlhausler B. S. (2011). Maternal “junk-food” feeding of rat dams alters food choices and development of the mesolimbic reward pathway in the offspring. *FASEB J.* 25 2167–2179 10.1096/fj.10-178392 21427213 PMC3114523

[B69] Papadimitriou A. Priftis K. N. (2009). Regulation of the hypothalamic-pituitary-adrenal axis. *Neuroimmunomodulation* 16 265–271 10.1159/000216184 19571587

[B70] Pecoraro N. Reyes F. Gomez F. Bhargava A. Dallman M. F. (2004). Chronic stress promotes palatable feeding, which reduces signs of stress: feedforward and feedback effects of chronic stress. *Endocrinology* 145 3754–3762 10.1210/en.2004-0305 15142987

[B71] Penke Z. Felszeghy K. Fernette B. Sage D. Nyakas C. Burlet A. (2001). Postnatal maternal deprivation produces long-lasting modifications of the stress response, feeding and stress-related behaviour in the rat. *Eur. J. Neurosci.* 14 747–755 10.1046/j.0953-816x.2001.01691.x 11556899

[B72] Peters A. Kubera B. Hubold C. Langemann D. (2011). The selfish brain: stress and eating behavior. *Front. Neurosci.* 5:74. 10.3389/fnins.2011.00074 PMC3105244 21660101

[B73] Pfenninger K. H. de la Houssaye B. A. Helmke S. M. Quiroga S. (1991). Growth-regulated proteins and neuronal plasticity. A commentary. *Mol. Neurobiol.* 5 143–151 10.1007/BF02935543 1823138

[B74] Proulx K. Clavel S. Nault G. Richard D. Walker C. D. (2001). High neonatal leptin exposure enhances brain GR expression and feedback efficacy on the adrenocortical axis of developing rats. *Endocrinology* 142 4607–4616 10.1210/endo.142.11.8512 11606425

[B75] Raspopow K. Abizaid A. Matheson K. Anisman H. (2010). Psychosocial stressor effects on cortisol and ghrelin in emotional and non-emotional eaters: influence of anger and shame. *Horm. Behav.* 58 677–684 10.1016/j.yhbeh.2010.06.003 20540943

[B76] Raspopow K. Abizaid A. Matheson K. Anisman H. (2014). Anticipation of a psychosocial stressor differentially influences ghrelin, cortisol and food intake among emotional and non-emotional eaters. *Appetite* 74 35–43 10.1016/j.appet.2013.11.018 24295926

[B77] Richard D. Lin Q. Timofeeva E. (2002). The corticotropin-releasing factor family of peptides and CRF receptors: their roles in the regulation of energy balance. *Eur. J. Pharmacol.* 440 189–197 10.1016/S0014-2999(02)01428-0 12007535

[B78] Rosmond R. Dallman M. F. Bjorntorp P. (1998). Stress-related cortisol secretion in men: relationships with abdominal obesity and endocrine, metabolic and hemodynamic abnormalities. *J. Clin. Endocrinol. Metab.* 83 1853–1859 10.1210/jc.83.6.1853 9626108

[B79] Rossi-George A. Virgolini M. B. Weston D. Cory-Slechta D. A. (2009). Alterations in glucocorticoid negative feedback following maternal Pb, prenatal stress and the combination: a potential biological unifying mechanism for their corresponding disease profiles. *Toxicol. Appl. Pharmacol.* 234 117–127 10.1016/j.taap.2008.10.003 18977374 PMC2656375

[B80] Rouach V. Bloch M. Rosenberg N. Gilad S. Limor R. Stern N. (2007). The acute ghrelin response to a psychological stress challenge does not predict the post-stress urge to eat. *Psychoneuroendocrinology* 32 693–702 10.1016/j.psyneuen.2007.04.010 17560728

[B81] Ryu V. Yoo S. B. Kang D. W. Lee J. H. Jahng J. W. (2009). Post-weaning isolation promotes food intake and body weight gain in rats that experienced neonatal maternal separation. *Brain Res.* 1295 127–134 10.1016/j.brainres.2009.08.006 19666012

[B82] Santana P. Akana S. F. Hanson E. S. Strack A. M. Sebastian R. J. Dallman M. F. (1995). Aldosterone and dexamethasone both stimulate energy acquisition whereas only the glucocorticoid alters energy storage. *Endocrinology* 136 2214–2222 10.1210/en.136.5.2214 7720670

[B83] Sapolsky R. M. Meaney M. J. (1986). Maturation of the adrenocortical stress response: neuroendocrine control mechanisms and the stress hyporesponsive period. *Brain Res.* 396 64–76 10.1016/0165-0173(86)90010-X 3011218

[B84] Sapolsky R. M. Romero L. M. Munck A. U. (2000). How do glucocorticoids influence stress responses? Integrating permissive, suppressive, stimulatory, and preparative actions. *Endocr. Rev.* 21 55–89 10.1210/edrv.21.1.0389 10696570

[B85] Savontaus E. Conwell I. M. Wardlaw S. L. (2002). Effects of adrenalectomy on AGRP, POMC, NPY and CART gene expression in the basal hypothalamus of fed and fasted rats. *Brain Res.* 958 130–138 10.1016/S0006-8993(02)03674-0 12468037

[B86] Shimizu H. Arima H. Watanabe M. Goto M. Banno R. Sato I. (2008). Glucocorticoids increase neuropeptide Y and agouti-related peptide gene expression via adenosine monophosphate-activated protein kinase signaling in the arcuate nucleus of rats. *Endocrinology* 149 4544–4553 10.1210/en.2008-0229 18535107

[B87] Spencer S. J. Tilbrook A. (2011). The glucocorticoid contribution to obesity. *Stress* 14 233–246 10.3109/10253890.2010.534831 21294656

[B88] Spencer S. J. Xu L. Clarke M. A. Lemus M. Reichenbach A. Geenen B. (2012). Ghrelin regulates the hypothalamic-pituitary-adrenal axis and restricts anxiety after acute stress. *Biol. Psychiatry* 72 457–465 10.1016/j.biopsych.2012.03.010 22521145

[B89] Stettler N. Stallings V. A. Troxel A. B. Zhao J. Schinnar R. Nelson S. E. (2005). Weight gain in the first week of life and overweight in adulthood: a cohort study of European American subjects fed infant formula. *Circulation* 111 1897–1903 10.1161/01.CIR.0000161797.67671.A7 15837942

[B90] Stevens A. Begum G. Cook A. Connor K. Rumball C. Oliver M. (2010). Epigenetic changes in the hypothalamic proopiomelanocortin and glucocorticoid receptor genes in the ovine fetus after periconceptional undernutrition. *Endocrinology* 151 3652–3664 10.1210/en.2010-0094 20573728

[B91] Strack A. M. Sebastian R. J. Schwartz M. W. Dallman M. F. (1995). Glucocorticoids and insulin: reciprocal signals for energy balance. *Am. J. Physiol.* 268 R142–R149 7840315 10.1152/ajpregu.1995.268.1.R142

[B92] Tanaka C. Asakawa A. Ushikai M. Sakoguchi T. Amitani H. Terashi M. (2009). Comparison of the anorexigenic activity of CRF family peptides. *Biochem. Biophys. Res. Commun.* 390 887–891 10.1016/j.bbrc.2009.10.069 19850009

[B93] Thomas M. B. Hu M. Lee T. M. Bhatnagar S. Becker J. B. (2009). Sex-specific susceptibility to cocaine in rats with a history of prenatal stress. *Physiol. Behav.* 97 270–277 10.1016/j.physbeh.2009.02.025 19268677

[B94] Vallee M. Mayo W. Dellu F. Le Moal M. Simon H. Maccari S. (1997). Prenatal stress induces high anxiety and postnatal handling induces low anxiety in adult offspring: correlation with stress-induced corticosterone secretion. *J. Neurosci.* 17 2626–2636 9065522 10.1523/JNEUROSCI.17-07-02626.1997 PMC6573515

[B95] Wang L. Stengel A. Goebel-Stengel M. Shaikh A. Yuan P. Q. Tache Y. (2013). Intravenous injection of urocortin 1 induces a CRF_2_ mediated increase in circulating ghrelin and glucose levels through distinct mechanisms in rats. *Peptides* 39 164–170 10.1016/j.peptides.2012.11.009 23183626 PMC3599411

[B96] Warne J. P. Akana S. F. Ginsberg A. B. Horneman H. F. Pecoraro N. C. Dallman M. F. (2009). Disengaging insulin from corticosterone: roles of each on energy intake and disposition. *Am. J. Physiol. Regul. Integr. Comp. Physiol.* 296 R1366–R1375 10.1152/ajpregu.91016.2008 19279289 PMC2689821

[B97] Warne J. P. Horneman H. F. Wick E. C. Bhargava A. Pecoraro N. C. Ginsberg A. B. (2006). Comparison of superior mesenteric versus jugular venous infusions of insulin in streptozotocin-diabetic rats on the choice of caloric intake, body weight, and fat stores. *Endocrinology* 147 5443–5451 10.1210/en.2006-0702 16873535

[B98] Weninger S. C. Muglia L. J. Jacobson L. Majzoub J. A. (1999). CRH-deficient mice have a normal anorectic response to chronic stress. *Regul. Pept.* 84 69–74 10.1016/S0167-0115(99)00070-1 10535410

[B99] Xu H. Hu W. Zhang X. Gao W. Liang M. Chen T. (2011). The effect of different maternal deprivation paradigms on the expression of hippocampal glucocorticoid receptors, calretinin and calbindin-D28k in male and female adolescent rats. *Neurochem. Int.* 59 847–852 10.1016/j.neuint.2011.07.010 21835217

[B100] Yakabi K. Noguchi M. Ohno S. Ro S. Onouchi T. Ochiai M. (2011). Urocortin 1 reduces food intake and ghrelin secretion via CRF_2_ receptors. *Am. J. Physiol. Endocrinol. Metab.* 301 E72–E82 10.1152/ajpendo.00695.2010 21540451 PMC3129836

[B101] Yura S. Itoh H. Sagawa N. Yamamoto H. Masuzaki H. Nakao K. (2005). Role of premature leptin surge in obesity resulting from intrauterine undernutrition. *Cell Metab.* 1 371–378 10.1016/j.cmet.2005.05.005 16054086

[B102] Zakrzewska K. E. Cusin I. Sainsbury A. Rohner-Jeanrenaud F. Jeanrenaud B. (1997). Glucocorticoids as counterregulatory hormones of leptin: toward an understanding of leptin resistance. *Diabetes* 46 717–719 10.2337/diab.46.4.717 9075817

[B103] Zakrzewska K. E. Cusin I. Stricker-Krongrad A. Boss O. Ricquier D. Jeanrenaud B. (1999). Induction of obesity and hyperleptinemia by central glucocorticoid infusion in the rat. *Diabetes* 48 365–370 10.2337/diabetes.48.2.365 10334315

